# Impact of P-Chloroaniline on Oxidative Stress and Biomacromolecules Damage in the Clam *Ruditapes philippinarums*: A Simulate Toxicity Test of Spill Incident

**DOI:** 10.3390/ijerph19095092

**Published:** 2022-04-22

**Authors:** Manni Wu, Jingjing Miao, Yuhan Li, Jiangyue Wu, Guoshan Wang, Dasheng Zhang, Luqing Pan

**Affiliations:** 1 Key Laboratory of Mariculture, Ministry of Education, Ocean University of China, Qingdao 266003, China; wumanni@stu.ouc.edu.cn (M.W.); jmiao@ouc.edu.cn (J.M.); wangqiaoqiao@stu.ouc.edu.cn (Y.L.); panlq@ouc.edu.cn (L.P.); 2 National Marine Hazard Mitigation Service, Ministry of Natural Resources of China, Beijing 100194, China; gswang@nmhms.org.cn; 3 Hebei Institute of Water Science, Shijiazhuang 050051, China; skyzhangdasheng@126.com

**Keywords:** p-chloroaniline, *Ruditapes philippinarums*, oxidative stress, DNA damage

## Abstract

As a hazardous chemical, p-chloroaniline (PCA) shows intensive adsorption and accumulation after entering the aquatic ecosystem, which can be enriched in organisms and cause damage. With the objective of achieving an integrated and mechanistic view of the toxic effects of PCA in the marine sentinel organism *Ruditapes philippinarum*, Manila clams were exposed to different concentration of PCA (0.5, 2 and 5 mg/L) for 15 days. Focusing on the gills, first targeting the toxic and digestive gland, the metabolic detoxification organ, we detected dose- and time-related changes inantioxidase activities and biomacromolecular damages in treated clams. Glutathione S-transferase (GST) activity and glutathione (GSH) contents were significantly induced, and superoxide dismutase (SOD) activity increased at the beginning of exposure and then decreased. The malondialdehyde (MDA) and protein methylation (PC) contents which represent lipid peroxidation and carbonylation of proteins, increased first with exposure time and then decreased in the digestive gland. DNA strand break levels were consistently higher than those in the control group. The digestive gland showed more sensitivity to the stress of PCA than the gills. GST and MDA in the gill and GST, GSH, SOD, DNA strand break level in the digestive gland showed significant correlation with PCA exposure, which indicated that these parameters can be used as sensitive biomarkers to indicate toxic effects from chloraniline leakage.

## 1. Introduction

Maritime transport of hazardous and noxious substances (HNS) is a significant manifestation of trade globalization and represents 11% of the chemicals traded worldwide [1]. A large number of maritime transport activities increase the risk of accidental spillage [2]. Additionally, HNS spills exhibit a wider range of behaviors (i.e., sinking, floating, gassing/evaporating, and dissolving) than oil spills, which would pose a huge threat to marine organisms and ecology as they indicate emergency and unpredictability [3,4]. However, currently not enough acute and chronic toxicological data for most priority HNS have been reported [5,6].

As an intermediate in various chemical manufacturing processes, p-chloroaniline (PCA) has been widely used in the manufacture of pigments, dyes, pharmaceuticals and pesticides. PCA, classified as a sinker (S) in the aquatic environment according to the European Behavior Classification System, is listed as a priority toxic pollutant in both US Environmental Protection Agency (EPA) and EU regulations [7]. When the amount of leakage is the same, a sinker is easier to disperse and accumulate, both in seawater and sediment [8,9,10]. At present, there are a few studies on the toxic effects of PCA, mainly on mammals and freshwater animals. Inaddition, PCA was a carcinogenic [11], embryotoxic [12], immunotoxic [13] and hematopoietic system and was also a target for PCA toxicity [14]. The 96 h lethal concentration 50% (LC_50_) values of PCA to *Lepomis macrochirus*, *Oncorhynchus mykiss* and *Gobiocypris rarus* were 2.4, 14.0 and 35.5 mg/L, respectively [15,16,17]. The 96 h concentration for 50% of maximal effect (EC50) value of PCA on *Nannochloropsis oculata*, on *Phaeodactylum tricornutu* and on *Platymonas subcordiformis* was 6.28, 10.00 and 13.05 mg/L, respectively [18,19,20]. *Danio rerio* exposed to PCA exhibits toxic effects in terms of hatching rate, deformity rate, mortality, growth, etc., while the changes inerythrocyte morphology suggested that the additional mode of action of PCA was respiratory disturbance [21]. Darlene et al. [22] found that PCA caused disorders in enzymes that were important for the health of *Oreochroms mossambcus*. Given that acute toxicity data for marine species is much less than for freshwater species and there are no chronic toxicity data for marine species (ECOTOX https://cfpub.epa.gov/ecotox/search.cfm, access on 5 March 2021), it is necessary to conduct acute and chronic toxicity studies of PCA to marine organisms.

The Manila clam *R. philippinarum* produced about four million tons worldwide in 2018, only less than *Crassostrea gigas* (FAO http://www.fao.org/fishery/statistics/en, access on 5 March 2021). As indicator organisms for marine and estuarine pollution monitoring, bivalves are regularly used as test species in eco-toxicological studies. Fluctuations in the response of different biomarkers to different toxicants provide a pattern of outcomes that can supply clues about the types of contaminants that cause the observed effects [23]. The effect of PCA on biomarkers in aquatic organisms is rarely studied. In this study, the acute toxicity of PCA to the Manila clam was evaluated. Furthermore, we exposed clams to PCA, and chose the gill and the digestive gland, which aremajor sites of xenobiotic uptake and metabolism foranalyzing the chronic toxicity effects of PCA to the Manila clam [24]. Glutathione S-transferase (GST) was selected as the detoxification enzyme implicated in hydroxy reactions of PCA. Glutathione (GSH), catalase (CAT) and superoxide dismutase (SOD) were chosen as antioxidant enzymes, together with the determination of malondialdehyde (MDA) content. Protein methylation level (PC content) and DNA damage level (F value) was also measured, in order to to determine the biomacromolecule damage caused by PCA exposure. The objective of this present study is to access the toxicity effect of PCA on bivalves, in order to provide basic data for biomarkers of ecological risk assessment and biomonitoring of PCA spills.

## 2. Materials and Methods

### 2.1. Animals

Manila clams *R. philippinarums* aged about 1 year old were sampled from Hong island of Qingdao and had a mean shell length of 2.15 ± 0.21 cm. The organisms were acclimated to lab conditions in 30 cm × 30 cm × 20 cm plastic aquarium for one week and the sea water was continuously aerated; mortality rate during this period was less than 5%. The temperature, dissolved oxygen and salinity of aquatic media during the whole experiment averaged 20.3 ± 1.2 °C, 6.8 ± 0.6 mg/L and 30‰, respectively.

### 2.2. Acute Toxicity Test

All the chemicals and reagents (analytical grade) used were supplied by Sigma (Hunterdon, NJ, USA). The PCA (CAS RN:106-47-8; 99% purity) was dissolved in dimethyl sulfoxide (DMSO) to prepare stock solution. DMSO was used because of its solubility and low toxicity and theamount used was <0.1% (*v/v*), which was constant in all treatments for a given experiment. The Manila clams were exposed to five concentrations of p-chloroaniline (20, 40, 60, 160 and 320 mg/L) plus two control (bland and solvent) after preliminary range-finding test. Three replicates were performed per treatment and each aquarium held 10 individuals. The test was static-renewal in which medium was renewed at 24 h intervals. The whole experiment was continuously inflated without feeding. Temperature, dissolved oxygen and salinity were kept the same as during the acclimation period. Dead organisms were removed immediately and mortalities were recorded at 24 h, 48 h, 72 h and 96 h. The shell was observed as open, and individuals with no response or very weak response to the adductor muscle after multiple stimuli was considered to be dead. The 96 h LC_50_ values and 95% confidence limits were calculated using a probit analysis method with SPSS (Ver. 24.0) software.

### 2.3. Chronic Toxicity Test

#### 2.3.1. Experimental Design

Exposure was implemented in a 30 cm × 30 cm × 20 cm plastic aquarium, with 60 mussels and 6 L seawater collected from Jiaozhou Bay per aquarium. PCA concentrations were selected based upon the 96 h LC_50_ (122.61 mg/L) values obtained in this study, and the report that the residual amount of simulated HNS leaking for 24 h is about 0.5 mg/L was provided by the State Oceanic Administration (SOA http://www.soa.gov.cn/, access on 5 March 2021). Organismsrepresenting five groups (control, solvent control, 0.5 mg/L, 2.0 mg/L and 5.0 mg/L) were considered. During the 15 days of experiment, the seawater was renewed every day. Each condition was executed in triplicate. After 0-, 1-, 3-, 8- and 15-day exposure, 10 individuals from each replicate were taken at every sampling time for chemical analyses. The day 0 control samples were selected randomly from each experimental aquarium for no difference between all groups before exposing to contaminants. The Manila clams were dissected, and the gill and digestive gland were then immediately excised, grinded and individually frozen at −80 °C until further analysis of the following biomarkers (GST, SOD, CAT activity, GSH, MDA content, PC content and DNA damage level).

#### 2.3.2. Measurement of Biomarkers

The protein content was determined according to the Coomassie brilliant blue G-250 method proposed by Bradford [25], and protein concentration was estimated by comparison with a standard solution of bovine serum albumin.

GST activity was determined by Habig et al. [26]. A unit of GST activity was defined as the number of glutathione conjugate formed using 1 nmol GSH and CDNB/min per mg protein.

GSH content was determined by Ellman [27]. It was calculated by the absorbance at 412 nm through a spectrophotomete. GSH is organized in µg/g protein.

SOD activity was measured according to Marklund and Marklund [28] with some modification at 440 nm. A unit enzyme activity was defined as the amount of enzyme required to inhibit the autoxidation rate of catechol per minute to 50% at 25 °C (U/mg protein).

CAT activity was monitored by the method presented by Greenwald [29] at 240 nm. The CAT activity of one unit was defined as 50% H_2_O_2_ consumption at 1 min (U/mg protein/min).

LPO levels, expressed by malondialdehyde (MDA) contents, were evaluated by an improved thiobarbituric acid method [30]. It was calculated by the absorbance at 530 nm through a spectrophotometer (UV-1800, Shimadzu, Kyotocity, Japan). Results were showed as nmol MDA generated/min/mg proteins.

PC contents were evaluated by following the method from Mecocci et al. [31]. Carbonyl content was measured from the absorbance of the solution at 370 nm. The results were expressed as nanomoles of DNPH binding/mg protein with a molar extinction coefficient of 22,000 M^−1^ cm^−1^.

DNA extraction and alkaline unwinding tests were carried out based on the work of Ching et al. [32]. The DNA samples were diluted and then divided into three equal types for fluorescence dsDNA, ssDNA and auDNA. The fluorescence of the samples was measured by a fluorescence spectrometer (Molecular Spectrum LS55, Waltham P.E., MA, USA) with the excitation wavelength of 360 nm and emission wavelength of 450 nm. A formula was used to calculate the ratio of double-stranded DNA to total DNA (F value): F value = (auDNA-ssDNA)/(dsDNA-ssDNA).

### 2.4. Data Analysis

Results of biochemical analysis were expressed as mean ± standard deviation (SD). All the data were analyzed using one-way ANOVA, which was followed by a multiple comparison of Duncan tests to describe significant differences. A probability level of less than 0.05 was considered statistically significant. The statistical computations were performed using SPSS (Ver. 24.0) software.

## 3. Results

### 3.1. Effect of P-Chloroaniline on the Activity of Detoxifying and Metabolizing Enzymes in R. philippinarum

**Figure 1:**
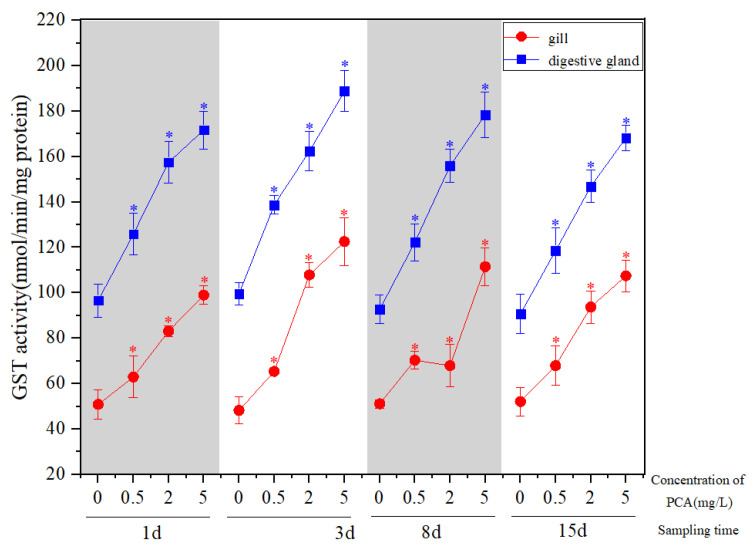
Effect of p-chloroaniline on GST activity in the gill and digestive gland of the Manila clam *Ruditapes philippinarum.* The results are presented as mean ± SD of three replicates. Asterisk indicates significant difference between the control and the p-chloroaniline-exposure group at the same sampling time. * *p* < 0.05.

P-chloroaniline stress can significantly induce GST activity in the gill and digestive gland of the Manila clam (Figure 1). The GST activity at 0.5 mg·L^−1^ was slightly higher than that of 2 mg·L^−1^ after 8 d exposure in the gill (*p* > 0.05). In addition, the GST activity of other experimental groups increased significantly with the increase inPCA concentration (*p* < 0.05). In the 2 mg·L^−1^ and 5 mg·L^−1^groups, PCA exposure in the gill reached the peak at 3 d (Figure 1). The GST activity of each treatment group in the digestive gland reached the peak at 3 d, and then showed a downward trend. At 15 d, the GST activity of each concentration group was still significantly higher than that of the natural seawater control group (*p* < 0.05) (Figure 1), and the activity in the digestive gland was higher than that in the gills.

### 3.2. Effect of P-Chloroaniline Stress on Antioxidant Defense System in R. philippinarum

**Figure 2:**
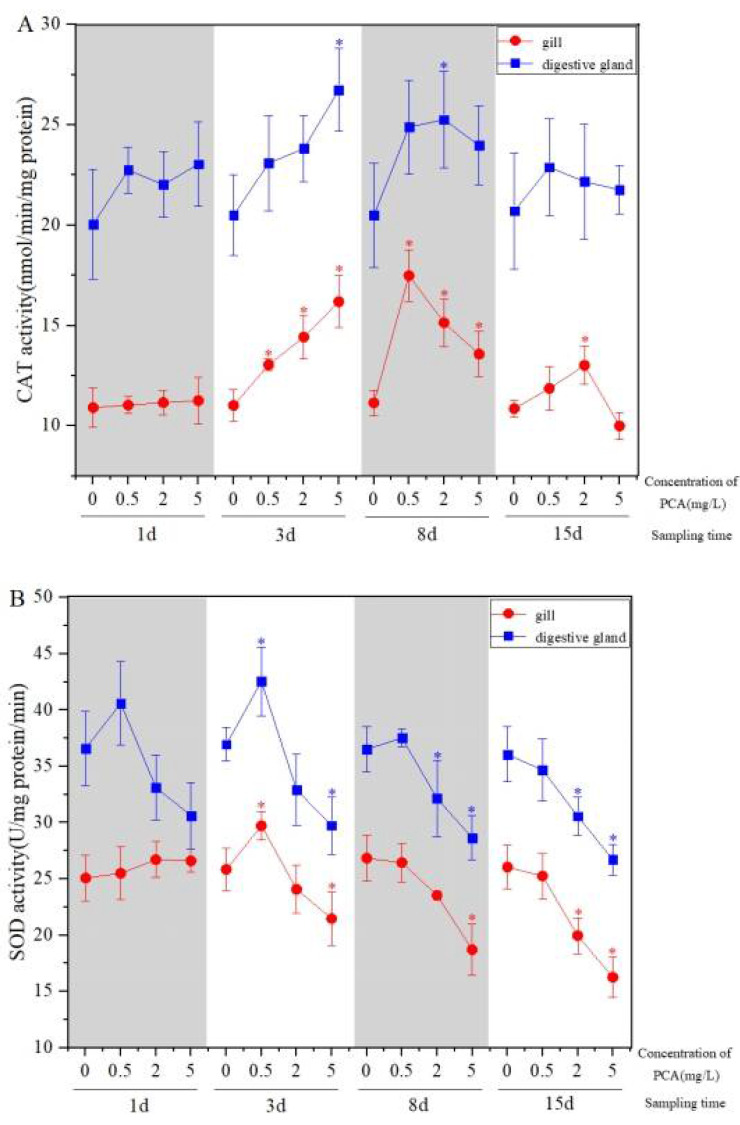

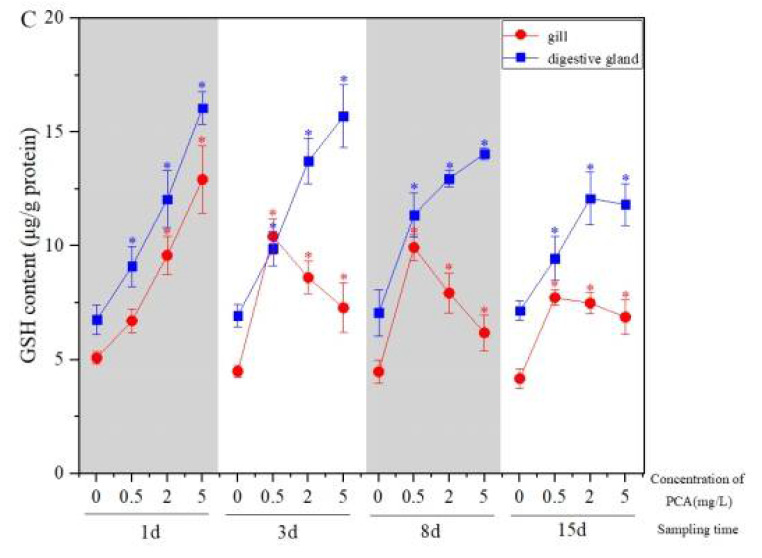
Effect of p-chloroaniline on CAT activity (**A**), SOD activity (**B**) and GSH content (**C**) in the gill and the digestive gland of the Manila clam *Ruditapes philippinarum*. The results are presented as mean ± SD of three replicates. Asterisk indicates significant difference between control and p-chloroaniline-exposure group at the same sampling time. * *p* < 0.05.

Statistically significant changes were observed in CAT and SOD activity and GSH content in the gill and digestive gland. Specifically, for the CAT activity in the gill and the digestive gland, there was no significant difference between the treatment group and the control group after exposure for 1 d (*p* > 0.05) (Figure 2A). The CAT activity increased with the increase inthis chemical concentration compared with the control after 3 d exposure, and the 5 mg·L^−1^ concentration group reached its maximum in both the gill and the digestive gland (*p* < 0.05). CAT activity, respectively reached its maximum in the 0.5 mg·L^−1^ and 2 mg·L^−1^ concentration groups after 8 d exposure. When exposed to 15 d, the CAT activity of each concentration group decreased, and the activity in the digestive gland was higher than that in the gills.

The SOD activity of each concentration group increased first and then decreased with the increase inPCA concentration in the gill and the digestive gland after 1 d and 3 d exposure, and decreased after 15 d exposure. The SOD activity of 0.5 mg·L^−1^ group reached the maximum value at 3 d, while that of 2 mg·L^−1^ and 5 mg·L^−1^ decreased with the increase of time. The activity in the digestive gland was higher than that in the gills (Figure 2B).

As shown in Figure 2C, compared with the control group of natural seawater, the content of GSH in the gill and the digestive gland of the Manila clam under p-chloroaniline stress was significant (*p* < 0.05). The GSH content in the gill increased significantly with the increase inconcentration after 1 d exposure (*p* < 0.05), 0.5 mg·L^−1^ group reached its maximum at 3 d exposure. The GSH content in the digestive gland increased with increasing concentration after 1–8 d exposure (*p* < 0.05).The peak value of low, medium and high concentration groups, respectively, appeared at 8 d, 3 d, 1 d, and the GSH content in the digestive gland was slightly higher than that in the gills.

### 3.3. Effect of P-Chloroaniline on Tissue Damage in R. philippinarum

**Figure 3:**
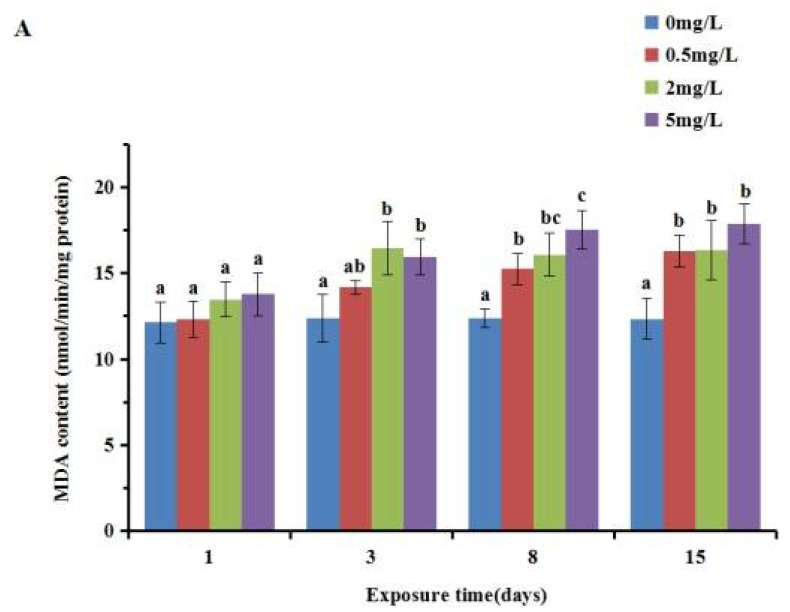

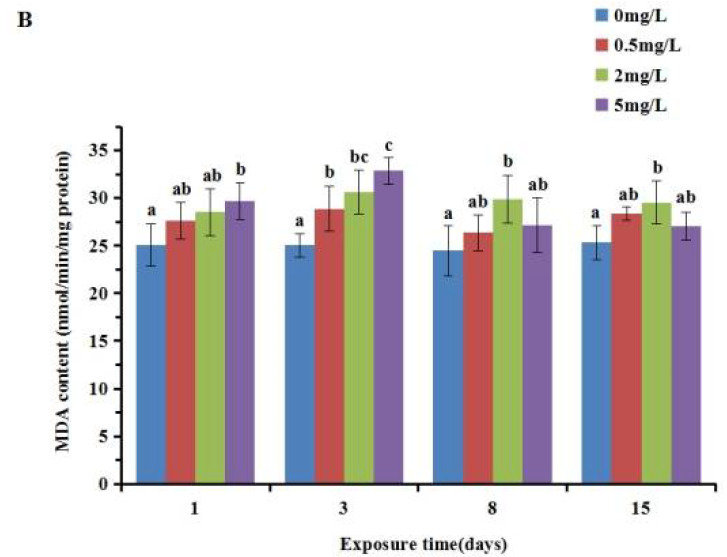
Effect of p-chloroaniline on MDA content in the gill (**A**) and digestive gland (**B**) ofthe Manila clam *Ruditapes philippinarum*. The results are presented as mean ± SD of three replicates. “a, b, c” means *p* > 0.05 if the letters are the same, and *p* < 0.05 if the letters are different.

The results of the analysis of lipid peroxidation showed that PCA had a significant impact on the MDA content, as shown in Figure 3. In the gill, there was an obvious time- and dose-dependent increase in all the p-chloroaniline exposure groups. A significant increase was observed between the experimental groups and the control group after 3, 8 and 15 d exposure (*p* < 0.05), whereas no significant difference (*p* > 0.05) was detected after exposure for 1 d. The content of MDA in the digestive gland increased gradually with the increase inconcentration after exposure for 1 and 3 d, but increased first and then decreased after 8 and 15 d of exposure. The concentration groups reached a peak after 3 d of exposure, and then decreased. The MDA content in the gill was lower than that in the digestive gland.

**Figure 4:**
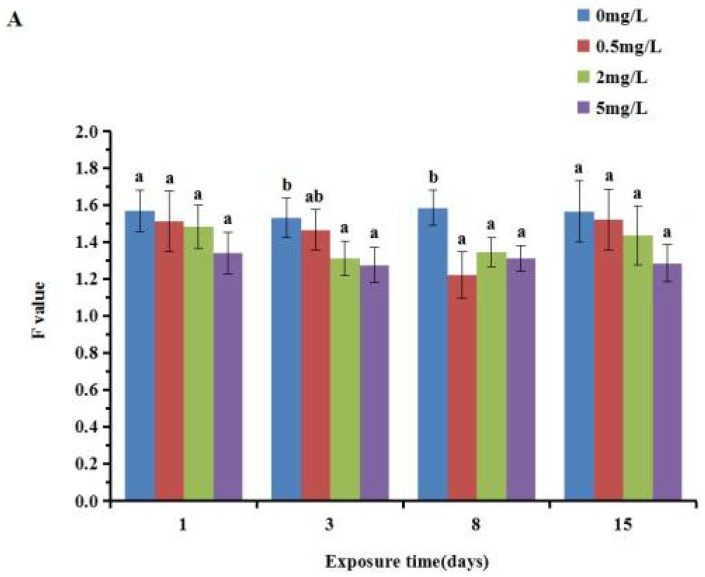

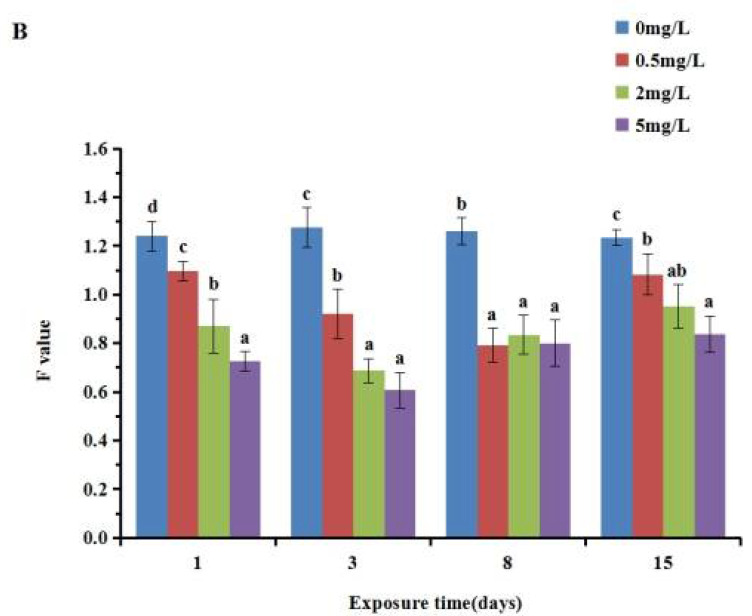
Effect of p-chloroaniline on F value in the gill (**A**) and digestive gland (**B**) of the Manila clam *Ruditapes philippinarum*. The results are presented as mean ± SD of three replicates. “a, b, c, d” means *p* > 0.05 if the letters are the same, and *p* < 0.05 if the letters are different.

The F value of each concentration group was lower than that of the control group, and it decreased first and then increased with the prolongation of time (Figure 4). In the gill, a significant decrease was detected after 3 and 8 d exposure (*p* < 0.05), whereas no significant difference (*p* > 0.05) was detected between the treated cultures and the control after exposure for 1 and 15 d. In the digestive gland, F value was found to be significantly decreased (*p* < 0.05) after exposure compared to the control group. The F value in the gill was slightly higher than that in the digestive gland.

**Figure 5:**
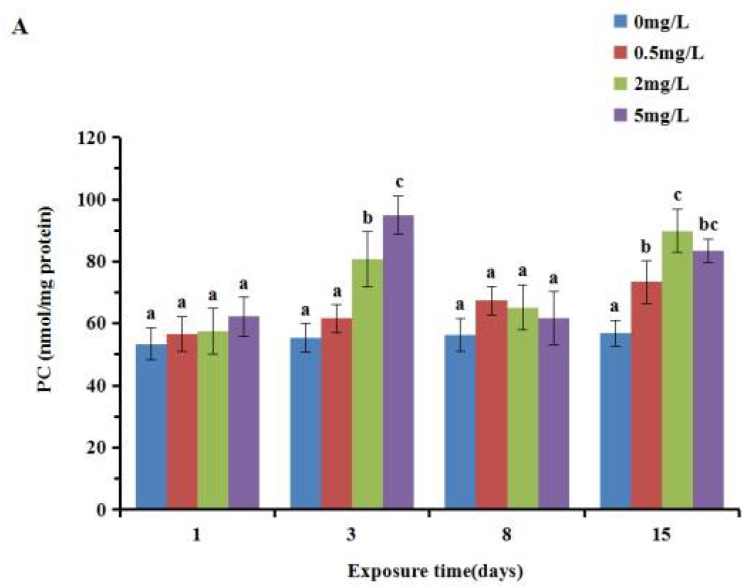

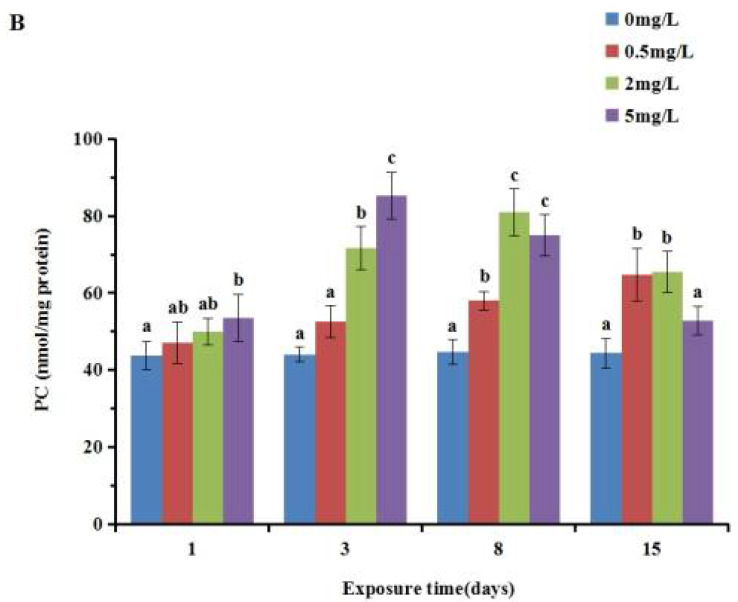
Effect of p-chloroaniline on PC content in the gill (**A**) and digestive gland (**B**) of the Manila clam *Ruditapes Philippinarum*.The results are presented as mean ± SD of three replicates. “a, b, c” means *p* > 0.05 if the letters are the same, and *p* < 0.05 if the letters are different.

As shown in Figure 5, the content of PC increased with the exposure concentration when the exposure times were 1 d and 3 d. At 8 d and 15 d after exposure, PC content first increased and then decreased with the concentration. In the gill, a significant increase (*p* < 0.05) was detected after 3 and 15 d exposure, whereas no significant difference (*p* > 0.05) was detected between the treated cultures and the control after exposure for 1 and 8 d. These variables showed an increasing trend (*p* < 0.05) in the digestive gland samples exposed to 2 and 5 mg/L p-chloroaniline concentrations for 3 and 8 d. The PC content in the gill was slightly higher than that in the digestive gland.

### 3.4. Correlation Analysis

The correlation analysis (Table 1) shows that, only the GST activity and lipid peroxidation level were positively correlated with the concentration of p-chloroaniline in the gill in Jiao Zhou Bay. GST activity, GSH content, DNA injury level and SOD activity were significantly correlated with the concentration of p-chloroaniline in the digestive gland, there was a significant positive correlation between GST and GSH, and the F value and SOD werenegatively correlated.

## 4. Discussion

### 4.1. Damage Mechanism of P-Chloroaniline on Biomacromolecules in R. philippinarum

When organisms are subjected to pollution stress, excessive accumulation of free radicals in the body can cause lipid peroxidation, while malondialdehyde (MDA) is the main product of lipid peroxidation in organisms, and its content can be used to represent the degree of lipid peroxidation. In recent years, it has been widely used in aquatic ecotoxicology. Many researchers have reported that heavy metals and other pollutants have toxic effects on marine life, leading to higher levels of MDA [33,34]. Long-term low-dose exposure of TBT can cause lipid peroxidation and cause body damage [35], MDA content increased with the increase inTBT exposure concentration. According to the results of this study, the content of MDA in the gills of the Manila clam increased after exposure to PCA, indicating that it caused lipid peroxidation in the body. In the digestive gland, MDA content in the 5 mg/L concentration group first increased with exposure time and then recovered to the level of the control group, indicating that the antioxidant system can defense the oxidative damage of pollutants to the Manila clam.

Protein carbonylation is one of the manifestations of oxidative stress caused by ROS in cells, tissues or organs [36], and PC can be used as a biomarker of oxidative stress [37]. Compared with other oxidation indicators, protein carbonylation is more advantageous due to its early and stable formation [38]. The PC content in the tissue of rare minnow (*Gobiocypris Rarus*) after exposure to hexabromocyclododecane (HBCD) showed an obvious time–dose effect [39]. Nile tilapia fish exposed to bifenthrin led to an increase inPC content in the liver [40]. In this study, with the increase inPCA exposure time, the PC content in the gill of the Manila clam presented a trend of induction–remediation–induction, while the biological species, pollutant types and environmental factors could influence the variation of proteincarbonyl content, leading to its difference from the experimental expectation.

When pollutants enter organisms, they form electron-philic active intermediates in the process of detoxification metabolism, which can directly combine with DNA molecules, produce a DNA adduct or lead to DNA strand break, finally causing oxidative damage to DNA [41]. DNA damage has been widely used as a biomarker for the toxicity assessment and environmental monitoring of pollutants. As proposed by Xu et al. [42], DNA damage to the gill and digestive gland of the Manila clam can be used as a biomarker for evaluating tetrabromodiphenyl ether contamination. Aniline has potential genotoxicity on mouse liver cells and haemocytes of black-spot frog (*Pelophylax nigromaculatus*), resulting in DNA damage [43]. In this study, DNA strand break occurred in the digestive gland from 1 day of exposure. The F value increased from 8 days of exposure in the medium and high concentration group, and increased from 15 days of exposure in the low concentration group, indicating that DNA damage repair occurred, and that the DNA repair time of the low concentration group was significantly later than that of the medium and high concentration groups. This is similar to the findings of [32]; we speculate that the repair mechanism is activated only when the damage caused by toxic exogenous substances exceeds a certain threshold.

### 4.2. Screening and Evaluation of Biomarkers of P-Chloroaniline Leakage

Biomarkers can directly reflect the interaction between exogenous toxic substances and cell molecules [44]. Different kinds or concentrations of exogenous toxic substances have different effects on the biochemical indexes of organisms, usually showing a certain dose–effect relationship, but some studies have found that pollutants of different concentrations have no significant effects on some biochemical indexes of organisms [45,46]. At present, there is no unified standard for the screening and evaluation of biomarkers at home and abroad. Oost et al. [47] took biomarkers that can be induced by pollutants, with a clear dose–effect relationship and stability as the evaluation criteria for their application. In this study, the toxic effects of PCA on various biomarkers in the gill and digestive gland of the Manila clam were measured, and the response trend of the biomarkers to PCA was analyzed with Pearson correlation, so as to provide a reference for screening sensitive biomarkers.

SOD, as the most critical first line of defense for scavenging reactive oxygen radicals in antioxidant enzymes, is often used as an indicator of oxidative stress to measure the stress of pollution on organisms [44]. Wang et al. [20] found that PCA could activate CAT and SOD enzymes of *Platymonas subcordiformis*. In this study, SOD activity was induced at low concentration and inhibited at high concentration, and finally all concentration groups were lower than the control group. The activity of CAT in the individual concentration group was lower than that in the control group. In addition, other groups are higher than the control group. The results showed that the two antioxidant enzymes in the Manila clam had a certain response to the stress of PCA, but the responses of SOD and CAT were different to some extent. This may be because they are the different functions of antioxidant enzymes. SOD can clear ROS in the body and catalyze oxygen ions to produce hydrogen peroxide, and CAT can break down hydrogen peroxide into oxygen ions. Their mechanism is different, thus contaminated stress may not show the same change rule. The results of this study are consistent with the normal response mechanism of organisms.

GSH is one of the non-enzymatic antioxidants and a cofactor of GST. When the oxidative stress in the organism exceeds a specific threshold, GSH cannot remove ROS, which will cause damage to the organism and reduce the activity of GST [48]. Tao et al. [49] showed that after 15 days of endosulfan exposure, GST activity and GSH content in the gill and the digestive gland of the Manila clam were significantly increased. In our study, there was a significant dose–effect relationship between GST activity and PCA concentration in the gill and digestive gland of the Manila clam, and GST activity was always induced. GSH content in the gill and the digestive gland was higher than that in the control group from the beginning to the end of the experiment, indicating that GSH was always induced, but the change trend of the two tissues was not completely consistent. This may be because the high concentration of PCA enhanced the activity of antioxidant enzymes in the gills, playing a major role in ROS elimination.

Based on the above results and combined with the index and the correlation analysis of the PCA concentration, we believe that GST activity in the gill and the digestive gland, GSH content and SOD activity in the digestive gland can be used as defensive biomarkers, and the MDA content of gills and F value of the digestive gland can be used as damage biomarkers, providing theoretical and technical support for PCA pollution leakage in the future.

## 5. Conclusions

The present study is the first to investigate the responses of bivalves exposed to sublethal concentrations of PCA which are comparable to contamination concentrations caused by real leakage. Changes in GST, SOD, CAT activity and GSH content showed the adaptive mechanism of Manila clams for reducing oxidative stress caused by PCA exposure. The DNA strand breakage, MDA and PC content indicated that PCA stress causes lipid peroxidation, protein carbonization and DNA damage, with some degree of recovery at the later stages of exposure. Most measured parameters changed more significantly in the digestive gland compared with gills, indicating that the digestive gland was more sensitive and vulnerable to the stress of PCA. The results of correlation analysis showed that GST activity, MDA content in the gill, GST activity, GSH content, SOD activity and F value in the digestive gland are promising biomarkers to reflect the harmful effects of PCA and used in the risk assessment of PCA leakage.

## Figures and Tables

**Table 1:** Correlation analysis between physiological indicators of the Manila clam *Ruditapes philippinarum* and p-chloroaniline concentration in Jiao Zhou Bay.

Organization	Index	*p* of Pearson’s Correlation Coefficient			
		1 d	3 d	8 d	15 d
gill	GST	0.963 **	0.965 **	0.970 **	0.957 **
	CAT	0.189	0.924 *	0.223	−0.122
	SOD	0.394	−0.618 *	−0.859 **	−0.907 **
	GSH	0.961 **	0.326	0.166	0.594 *
	MDA	0.582 *	0.773 *	0.891 **	0.808 **
	PC	0.525	0.934 **	0.233	0.730 **
	F value	−0.592 *	−0.768 **	−0.502	−0.638 *
Digestivegland	GST	0.962 **	0.979 **	0.978 **	0.974 **
	CAT	0.462	0.783 **	0.450	0.128
	SOD	−0.620 *	−0.665 *	−0.810 **	−0.886 **
	GSH	0.971 **	0.967 **	0.926 **	0.872 **
	MDA	0.675 *	0.866 **	0.460	0.345
	PC	0.675 *	0.964 **	0.864 **	0.301
	F value	−0.957 **	−0.936 **	−0.775 **	−0.926 **

Note: MDA indicates lipid peroxidation level; PC indicates protein methylation level; F value indicates DNA injury level; * indicates significant correlation (*p* < 0.05); ** indicates extremely significant correlation (*p* < 0.01).

## Data Availability

The data are available upon demand by request to the corresponding author.
